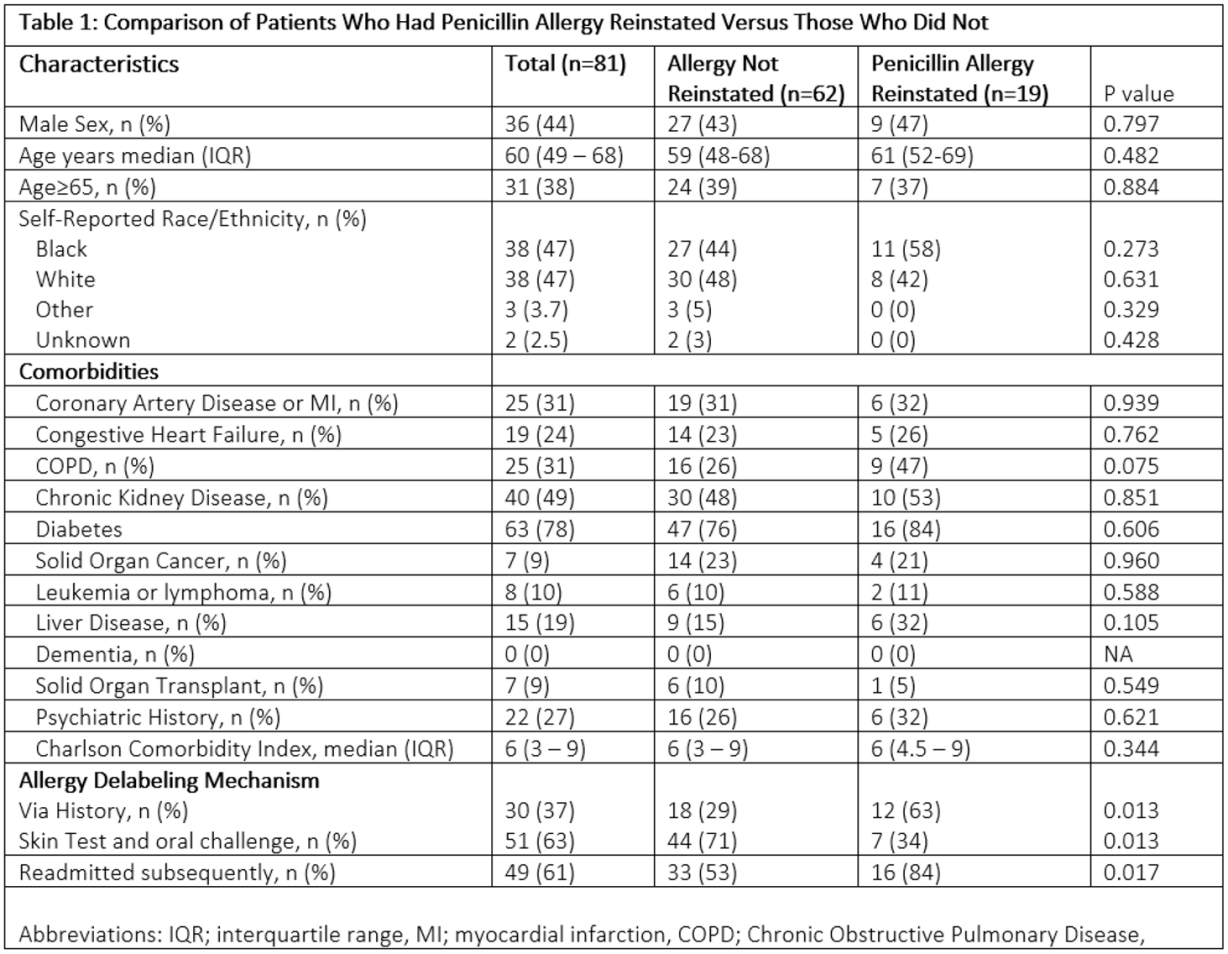# Penicillin allergy reinstatement in a cohort of patients previously delabeled following formal allergy assessment

**DOI:** 10.1017/ash.2022.95

**Published:** 2022-05-16

**Authors:** Lea Monday, Ravitej Goteti, Jaclyn Michniak, Edward Zoratti, Allison Weinmann

## Abstract

**Background:** Penicillin allergies are frequently reported and are associated with adverse clinical and antimicrobial stewardship outcomes. Allergy delabeling, either by patient history or skin testing and oral challenge can facilitate removal of penicillin allergy label. However, penicillinallergies are often reinstated in the medical record and data is limited about how and why this occurs. In our center, the departments of allergy and infectious diseases utilize an allergist nurse practitioner for penicillin allergy delabeling. We investigated the prevalence of penicillin allergy reinstatement following removal and associated factors thereof. **Methods:** We performed a retrospective observational study of patients who previously had penicillin allergy removed by the allergist nurse practitioner between August 2020 and May 2021 (250 days). Patients were followed for a minimum of 8 months and up to 16 months after penicillin allergy removal. We then assessed whether the allergy was reinstated. Clinical characteristics were compared between patients with penicillin allergy reinstated and not reinstated using the χ^2^ and Mann-Whitney *U* test. The primary end point was prevalence of penicillin allergy reinstatement following removal. **Results:** During the study period, 81 patients had penicillin allergy removed, but it was later reinstated in 19 patients (23%) (Fig 1). Median time to reinstatement was 94 days. Allergies were reinstated most frequently by nurses (53%) and medical assistants (37%). Reinstatement occurred in both outpatient (53%) and inpatient (47%) settings. In 18 of 19 cases, there was no acknowledgment that a prior assessment had determined the patient was not allergic to penicillin. Only 1 patient experienced a reaction prompting reinstatement of penicillin allergy. Once the allergy was redocumented, it was subsequently mentioned in a median of 17 notes per patient. Comorbidities did not differ between patients with allergy reinstated versus those without (Table 1). Patients with penicillin allergy reinstated were more often originally delabeled via history rather than skin test followed by oral challenge and were more likely to have been readmitted subsequently. **Conclusions:** Penicillin allergies were redocumented in almost one-quarter of patients, most frequently by a nonphysician team member and without acknowledgement of prior removal. Patients who undergo skin testing may be less likely to continue to report a penicillin allergy to medical staff compared to those whose allergy is removed based on history. Increased interactions with the healthcare system may have contributed to having the allergy reinstated.

**Funding:** None

**Disclosures:** None

**Figure:**
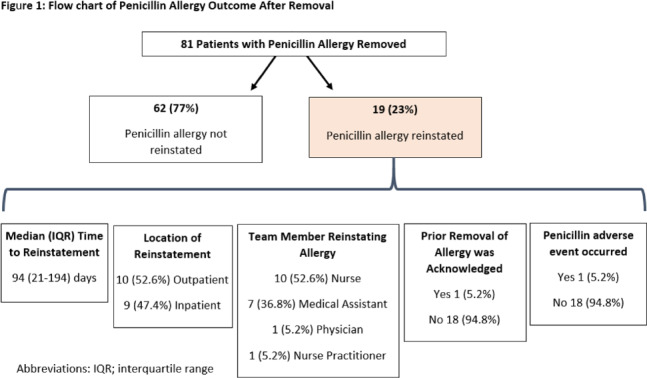

**Table:**